# Supplementary figures and images for: An improved method for preparing stained ground teeth sections
Source: PeerJ. 2023 Apr 28;11:e15240. doi: 10.7717/peerj.15240 (PMC10150715; doi:10.7717/peerj.15240)

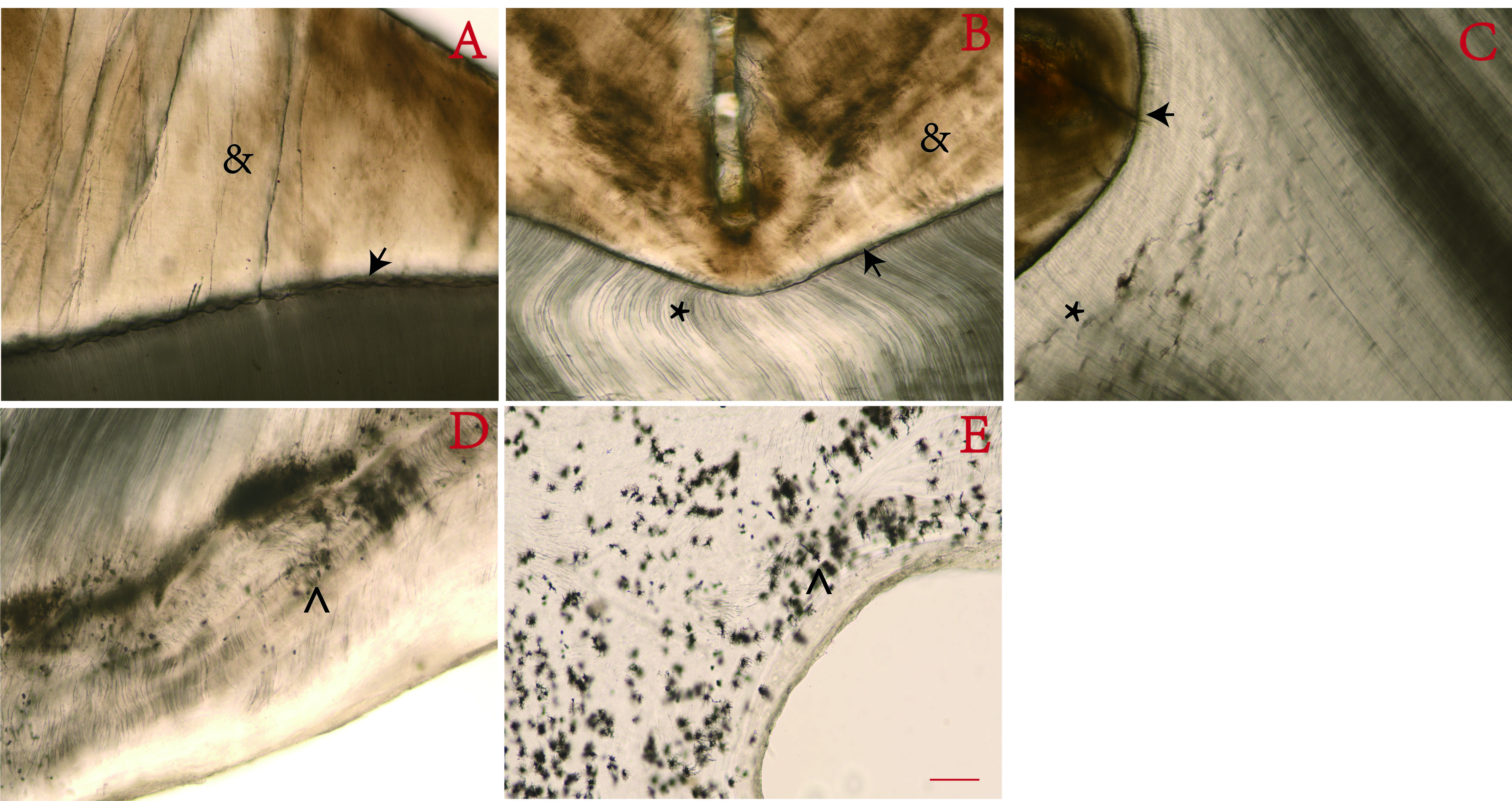

Supplement: Figure S1 — Scale bar = 100 µm. [file peerj-11-15240-s001.jpg]
